# The Nuclear DNA Content and Genetic Diversity of *Lampetra morii*

**DOI:** 10.1371/journal.pone.0157494

**Published:** 2016-07-07

**Authors:** Xinyu Yan, Wenbin Meng, Fenfang Wu, Anlong Xu, Shangwu Chen, Shengfeng Huang

**Affiliations:** 1 State Key Laboratory of Biocontrol, Guangdong Key Laboratory of Pharmaceutical Functional Genes, School of Life Sciences, Sun Yat-sen University, Guangzhou, People’s Republic of China; 2 Beijing University of Chinese Medicine, Beijing, People’s Republic of China; Laboratoire Arago, FRANCE

## Abstract

We investigated the nuclear DNA content and genetic diversity of a river lamprey, the Korean lamprey *Lampetra morii*, which is distributed in the northeast of China. *L*. *morii* spends its whole life cycle in fresh water, and its adult size is relatively small (~160 mm long) compared with that of other lampreys. The haploid nuclear DNA content of *L*. *morii* is 1.618 pg (approximately 1.582 Gb) in germline cells, and there is ~15% germline DNA loss in somatic cells. These values are significantly smaller than those of *Petromyzon marinus*, a lamprey with a published draft genome. The chromosomes of *L*. *morii* are small and acrocentric, with a diploid modal number of 2n = 132, lower than some other lampreys. Sequence and AFLP analyses suggest that the allelic polymorphism rate (~0.14% based on examined nuclear and mitochondrial DNA sequences) of *L*. *morii* is much lower than that (~2%) of *P*. *marinus*. Phylogenetic analysis based on a mitochondrial DNA fragment confirms that *L*. *morii* belongs to the genus *Lampetra*, which, together with the genus *Lethenteron*, forms a sister group to *P*. *marinus*. These genetic background data are valuable for subsequent genetic and genomic research on *L*. *morii*.

## Introduction

Lampreys are modern representatives of the jawless vertebrate lineage that diverged from the jawed vertebrate lineage some 500 million years ago [1]. In addition to occupying an important phylogenetic position, lampreys possess a unique adaptive immune system that is based on variable lymphocyte receptors rather than antibodies [2, 3]. Therefore, lampreys can be of both evolutionary and immunological interest. There are more than 40 lamprey species separated into three families, including Petromyzonidae, Mordaciidae and Geotriidae [4]. Petromyzonidae includes the most species and is distributed in the northern hemisphere, whereas the other two families live in the southern hemisphere [4].

Robinson et al. evaluated the DNA values for somatic (erythrocyte) 2C nuclei of nine different lamprey species, which lie between 2.2 and 2.6 pg, with *Petromyzon marinus* as an exception (3.6 pg) [5]. Recently, it has been shown that the estimated DNA content of germline (sperm) 2C nuclei in *P*. *marinus* is approximately 4.6 pg, which is ~20% higher than that of the somatic nuclei [6]. This deficit turns out to be due to programmed genome rearrangement during early embryogenesis in *P*. *marinus*, which results in approximately 20% germline DNA loss in somatic tissues [6–8]. Germline DNA loss has also been observed in somatic cells in hagfish, another lineage of jawless vertebrates [9]. Karyotype has been analyzed in lamprey species from all three families [4, 10–12]. The chromosomes of Petromyzonidae and Geotriidae are mostly acrocentric and range between 0.5–5 μm, with a modal diploid number of 154–178 [13], whereas the chromosomes of Mordaciidae are mainly metacentric or submetacentric with a modal diploid number of 76, probably due to chromosome fusions [11, 14]. The genomes of both northern and southern hemisphere lamprey species exhibit high GC content, as revealed by high levels of four-fold degenerate sites (70–90%) [15, 16]. Different molecular markers have been developed in lamprey for population and evolutionary genetic studies. Filcek et al. utilized 7 microsatellite markers to evaluate the genetic variation between four lamprey species, identifying lamprey larvae to species [17]. Mejia et al. developed 88 random amplification polymorphic DNA (RAPD) markers in the Mexican lamprey *Lampetra geminis* and observed a low heterozygosity of 0.21–0.25 [18]. It is also reported that the outbred sea lamprey *P*. *marinus* exhibits high sequence disparity (~2%) between alleles at most genomic loci [19].

The complete mitochondrial genomes of several lamprey species have been published. The gene order and contents of lamprey mitochondrial genomes are basically identical to those of other chordate mtDNAs, which indicates that the common vertebrate mitochondrial gene organization had already been established before the split of jawless and jawed vertebrates [20–23]. The nuclear genome of the sea lamprey *P*. *marinus* was sequenced and assembled; it was generated from liver cells and thus does not include the ~20% germline genomic content [19]. This draft genome spans 0.816 Gb, which is approximately half of the estimated haploid somatic genome size (1.76 Gb), and exhibits high proportions of repetitive elements and GC content [19]. The draft genome of the Japanese lamprey, *Lethenteron japonicum*, has also been published, and it spans 1.03 Gb [24].

There are three lamprey species distributed in the northeast of China, *Lampetra morii*, *Lethenteron reissneri* and *Lethenteron japonicum*, and they are often used for research. Here, we profiled the overall genetic and genomic characteristics of *L*. *morii*. We showed that *L*. *morii* has a relatively small genome, fewer chromosomes and lower heterozygosity compared to *P*. *marinus* and other lampreys.

## Results

### The nuclear DNA content of *L*. *morii*

**Fig 1:**
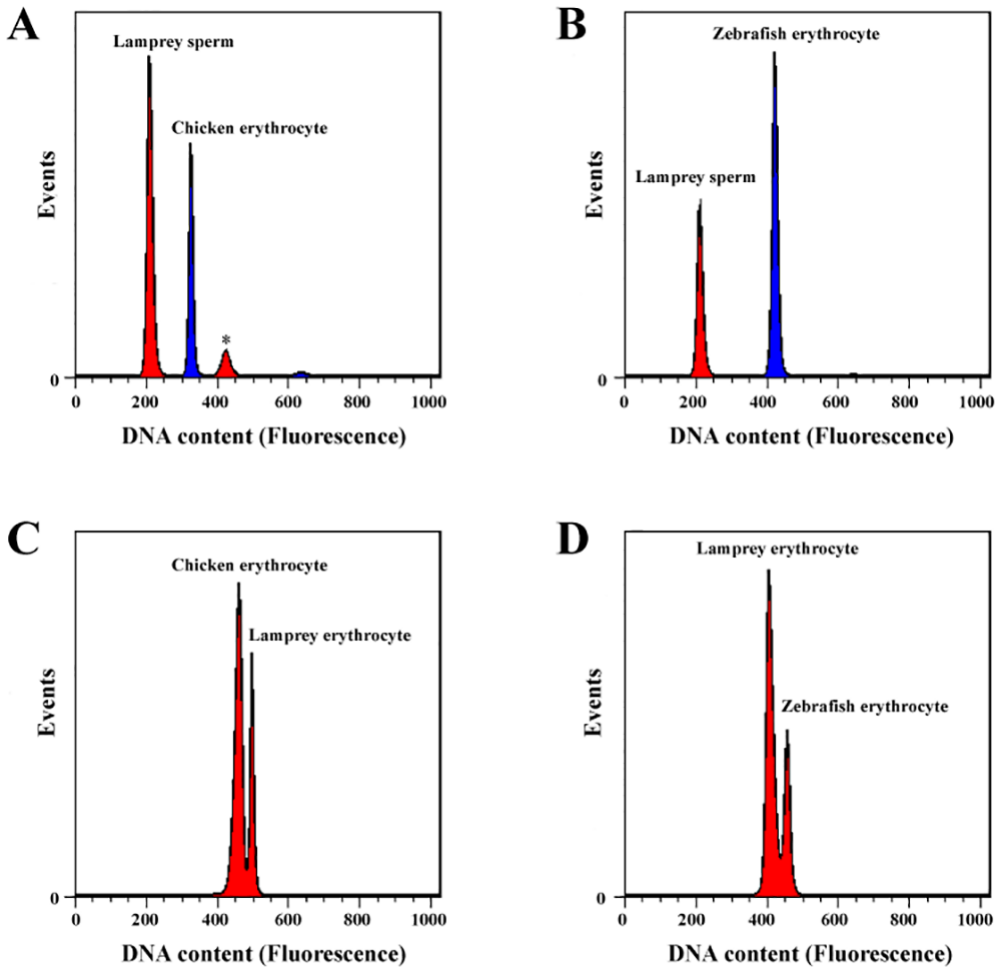
The nuclear DNA content of *L*. *morii*. Nuclei from each tissue were isolated from the same three individuals, propidium iodide-stained and analyzed by flow cytometry. Chicken erythrocytes (2C = 2.50 pg DNA) and zebrafish erythrocytes (2C = 3.223 pg DNA) served as independent internal reference standards. (A, B) Sperm nuclear DNA content with chicken erythrocytes (A) and zebrafish erythrocytes (B). An asterisk marks a peak corresponding to spermatogonia or sperm doublets. (C, D) Erythrocyte nuclear DNA content with chicken erythrocytes (C) and zebrafish erythrocytes (D). The horizontal axis represents the fluorescence value of cells, which is directly proportional to DNA content within the same sample. The vertical axis represents the number of single cells detected.

Using flow cytometry methods with chicken erythrocytes (2C = 2.50 pg DNA) as well as zebrafish erythrocytes (2C = 3.22 pg DNA) as internal reference standards, the nuclear DNA content of different tissues of *L*. *morii* was evaluated, the values of which were highly reproducible across individuals and preparations. Our result shows that the DNA content of different tissues is not always the same in *L*. *morii*. The DNA content of sperm nuclei (1C = 1.618 ± 0.001 pg) (Fig 1A and 1B), which represents germline tissue, is almost 15% more than that of somatic tissues such as erythrocytes (1C = 1.376 ± 0.026 pg), gill (1C = 1.385 ± 0.009 pg), kidney (1C = 1.406 ± 0.012 pg), and heart (1C = 1.402 ± 0.015 pg) (Fig 1C and 1D). This coincides with findings in *P*. *marinus* that the genome size of the germline is >20% larger than soma [6]. The somatic DNA content of *L*. *morii* is close to that of most lampreys (2C = 2.2–2.6 pg) but 23% less than *P*. *marinus'* (2C = 3.6 pg) [6]. The germline genome size of *L*. *morii*, as inferred from the nuclear DNA content, is 1.582 Gb (haploid), which is relatively small among vertebrates.

### Karyotype analysis

**Fig 2:**
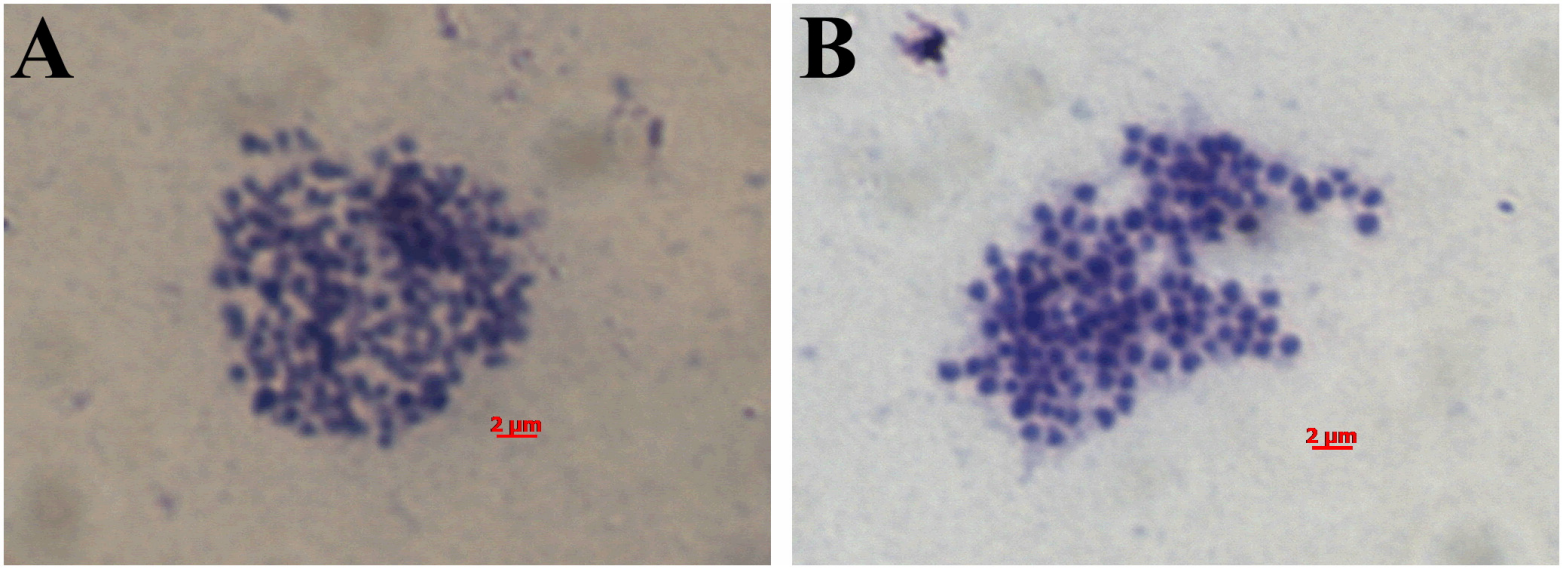
Karyotype analysis of *L*. *morii*. Colchicine was injected into adult *L*. *morii* to arrest cell division at metaphase. The gills and intestine were separated, Giemsa stained and observed through high magnification microscopy. The gill (A) and intestine (B) chromosome numbers were 2n = 132 and 2n = 131, respectively. Two more individuals were tested, and the chromosome numbers ranged from 130 to 132.

To analyze the chromosomal characteristics of *L*. *morii*, we injected colchicine into adult fish to obtain single metaphase cells, followed by Giemsa staining and microscopic observation. Analysis of three gill karyotypes and three intestine karyotypes from the same individual gave a modal number of 2n = 132 chromosomes (Fig 2A and 2B). Preparation artifacts caused by the high number of small-sized chromosomes might have led to small differences between the tests. The chromosome number of *L*. *morii* is lower than that of both *P*. *marinus* (2n = 164) [25] and almost all of the northern hemisphere lamprey species whose chromosomes have been counted [4, 13]. The chromosomes of *L*. *morii* were found to be approximately 0.5 μm, similar to other Petromyzonidae species [13], but no bi-armed chromosomes were found under high magnification oil microscopy, suggesting no obvious chromosome fusion events.

### AFLP profiles

**Fig 3:**
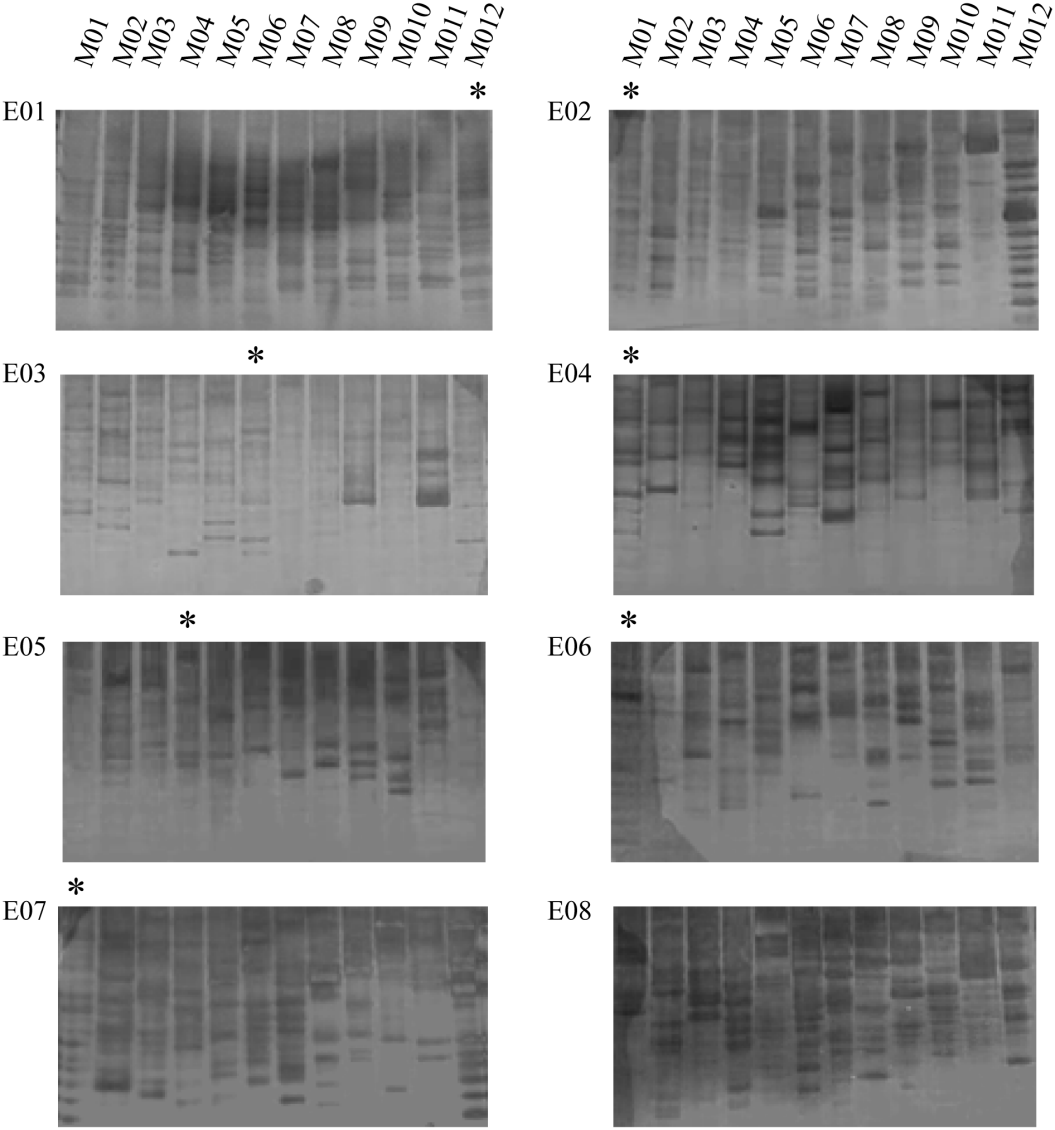
Amplification of 96 pairs of *Eco*RI-*Mse*I fragments. According to the number, clarity and distribution range of the bands, we screened 7 primer combinations (with an asterisk) to analyze polymorphism: E01/M12 (*Eco*RI+AAC/*Mse*I+CCT), E05/M04 (*Eco*RI+AGA/*Mse*I+CAT), E02/M01 (*Eco*RI+AAC/*Mse*I+CAA), E06/M01 (*Eco*RI+AGT/*Mse*I+CAA), E03/M06 (*Eco*RI+ACA/*Mse*I+CCT), E07/M01 (*Eco*RI+ATC/*Mse*I+CAA), E04/M01 (*Eco*RI+ACT/*Mse*I+CAA). The primer pairs are listed in Table 2. The molecular weight size range of the fingerprints is from 20 to 500 nucleotides.

To generate AFLP fingerprints for *L*. *morii*, we followed the protocol described by Vos et al. [26] in 1995 to design and screen primer pairs. According to band numbers and the clarity and distribution range of each band (Fig 3), seven primer combinations were selected to analyze polymorphism in *L*. *morii*, and five randomly chosen *L*. *morii* individuals were used. For each of the seven primer combinations, the total number of bands and polymorphism bands ranged from 92–122 and 9–16, respectively (Table 1), and was very close between primer pairs. The average AFLP rate of *L*. *morii* was found to be 11.83%, which is lower than that of cattle [27].

**Table 1:** AFLP polymorphism rates for *L*. *morii*.

Primer combination	Samples	Total number of bands	Polymorphism	
			bands	rate (%)
E01/M12	5	116	15	12.93
E05/M04	5	92	9	9.78
E02/M01	5	110	14	12.73
E06/M01	5	108	11	10.19
E03/M06	5	117	13	11.11
E07/M01	5	113	14	12.39
E04/M01	5	122	16	13.11
Total	35	778	92	11.83

**Table 2:** Adapters and primers used in AFLP analysis.

Adapters/primers	Sequence
*Eco*RI adapter	5′-CTCGTAGACTGCGTACC-3′
	3′-CATCTGACGCATGGTTAA-5′
*Mse*I adapter	5′-GACGATGAGTCCTGAG-3′
	3′-TGCTACTCAGGACTCAT-5′
Primers *Mse*I	
M01	5′-GATGAGTCCTGAGTAACAA-3′
M04	5′-GATGAGTCCTGAGTAACAT-3′
M06	5′-GATGAGTCCTGAGTAACCT-3′
M12	5′-GATGAGTCCTGAGTAACTT-3′
Primers *Eco*RI	
E01	5′-GACTGCGTACCAATTCAAC-3′
E02	5′-GACTGCGTACCAATTCAAG-3′
E03	5′-GACTGCGTACCAATTCACA-3′
E04	5′-GACTGCGTACCAATTCCAT-3′
E05	5′-GACTGCGTACCAATTCCCA-3′
E06	5′-GACTGCGTACCAATTCCCT-3′
E07	5′-GACTGCGTACCAATTCCGA-3′

### SNP analysis of selected gene fragments

We chose five housekeeping genes, including actin-like 6B (*ACTL6B*), glyceraldehyde-3-phosphate dehydrogenase (*GAPDH*), heat shock protein (*HSP*), phosphoglycerate kinase 1 (*PGK1*) and ribosomal protein L32 (*RPL32*), to analyze the SNP rates in *L*. *morii*. The amphioxus *Branchiostoma belcheri* was used for comparison. For each gene, one intron with a suitable length was amplified via PCR. Three isolated adult individuals from each of *L*. *morii* and *B*. *belcheri* were tested. The polymorphism rate for SNPs in *B*. *belcheri* was 9.327% for the limited samples and the selected fragment, consistent with the 5–15% SNP rates in the outbred amphioxus [28]. The rate in *L*. *morii* was 0.145% based on the examined intronic sequences (Table 3), which was far lower than *B*. *belcheri*. In addition, we found that the indels of *L*. *morii* were fewer and shorter than those of *B*. *belcheri*. These data suggest that the heterozygosity of *L*. *morii* is much lower than the genome-wide average allelic polymorphism rate (~2%) of *P*. *marinus* [29].

**Table 3:** SNPs in five selected introns of *L*. *morii* and *B*. *belcheri*.

Species	Gene	Length (bp)	SNP	Indel	Polymorphism rate for SNPs (%)	Average rate for SNPs (%)
*L*. *morii*	*ACTL6B*	800	1	1	0.125	0.145
	*GAPDH*	531	1	0	0.188	
	*HSP*	551	1	0	0.181	
	*PGK1*	795	1	1	0.126	
	*RPL32*	950	1	0	0.105	
*B*. *belcheri*	*ACTL6B*	552	20	3	3.623	9.327
	*GAPDH*	858	123	21	14.336	
	*HSP*	1398	17	2	1.216	
	*PGK1*	554	74	14	13.357	
	*RPL32*	546	77	13	14.103	

### Noncoding region of the *L*. *morii* mitochondrial genome

**Fig 4:**
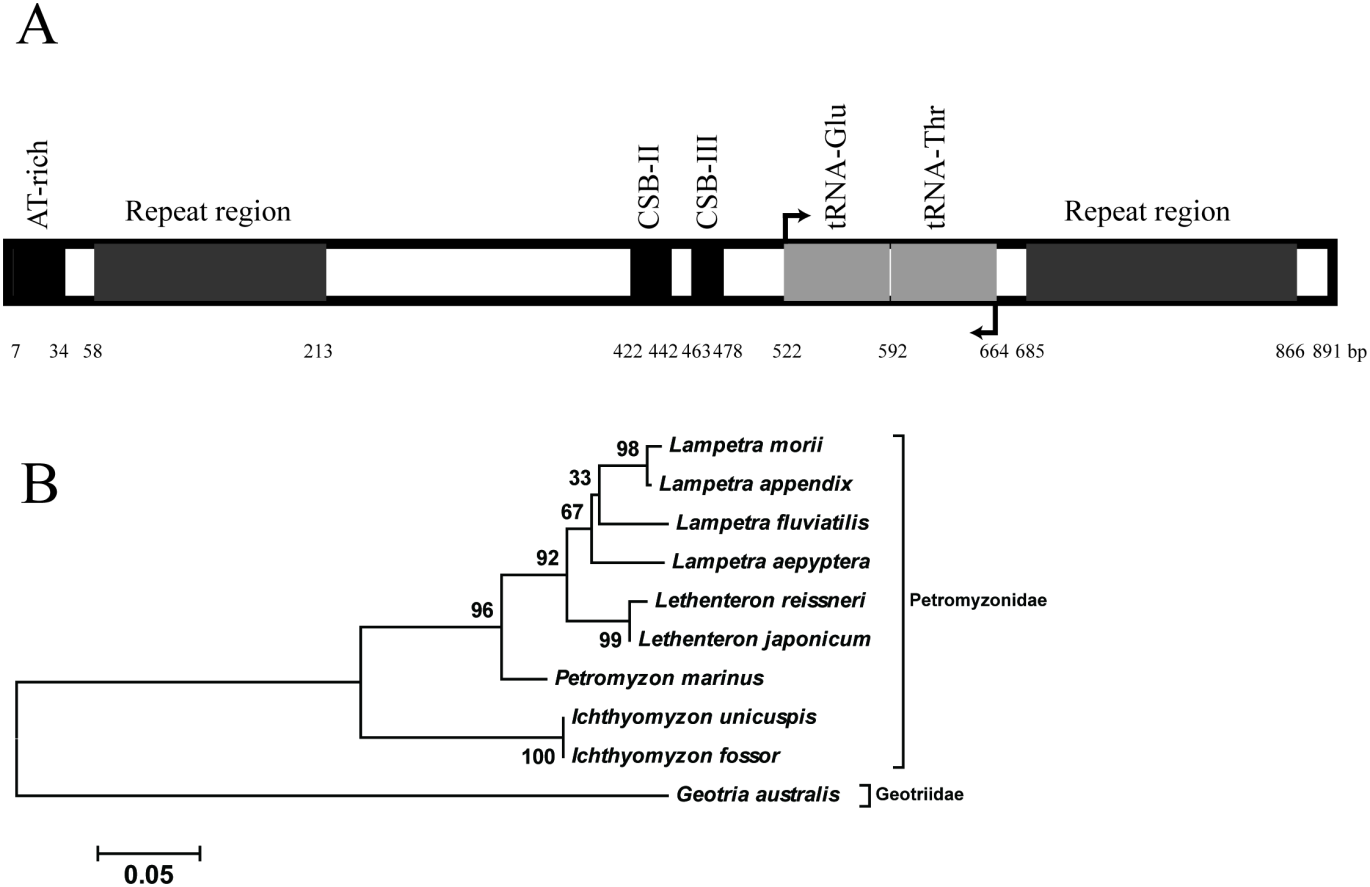
Structure of the *L*. *morii* mitochondrial noncoding region (A) and a phylogenetic tree based on this region (B). The tree was constructed using the maximum likelihood method with the Tamura—Nei substitution model. The mitochondrial noncoding region sequences of *P*. *marinus*, *Lethenteron reissneri*, *Lethenteron japonicum*, *Lampetra fluviatilis*, *Lampetra aepyptera*, *Lampetra appendix*, *Ichthyomyzon unicuspis*, *Ichthyomyzon fossor*, and *Geotria australis* were obtained from the NCBI database with accession numbers NC_001626.1, KC353466.1, KC353468.1, Y18683.1, NC_026917.1, NC_025583.1, NC_025553.1, NC_025552.1, and NC_029404.1, respectively.

We amplified the noncoding region of *L*. *morii* mitochondria genome via PCR, which is flanked by the NADH dehydrogenase 6 *(ND6*) and cytochrome b (*CYTB*) genes. This noncoding region was found to be 891 bp in length and separated by the *tRNA-Thr* and *tRNA-Glu* genes (Fig 4A), coinciding with early findings in other lamprey species [20–23, 30]. *L*. *morii* contains four tandem copies of a 39 bp repeat (5′-TATGCCTCTATGGCATAGGTATATATAATGACATAGGTA-3′), more than the three copies in *P*. *marinus* [22]. A 26 bp repeat region is the most diverse region of the *L*. *morii* mitochondria genome and is composed of 7~9 tandem repeat copies (5′-TTGTAATTTTAAAATTTCTTTTTTAA-3′), coupled with SNPs and small indels between copies. The conserved sequence block, CSB-II and CSB-III, is conserved with other lamprey species. The SNP rate of the noncoding region of *L*. *morii* mitochondria, excluding the 26 bp repeat region, was found to be 0.146%, a very low level and consistent with the above estimation with nuclear gene fragments. A phylogenetic tree was constructed with other lamprey mitochondria noncoding regions (Fig 4B), and it suggests a closer relationship among *Lampetra*, *Lethenteron* and *Petromyzon*.

## Discussion

Lamprey is an important model organism for evolutionary and immunological studies. Analysis of its noncoding mitochondrial sequence confirms that *L*. *morii* is related to *P*. *marinus* and *L*. *japonicum*, both of which have a published draft genome [24, 29]. Similar to *P*. *marinus* [6], the genetic material of *L*. *morii* had experienced germline DNA loss in soma (approximately 15% loss). The genome size of *L*. *morii* is similar to that of *L*. *japonicum* (~1.6 Gb) but is only ~70% of *P*. *marinus'*, suggesting a potentially large difference between the genomes of *L*. *morii* and *P*. *marinus*. The modal number of *L*. *morii* chromosomes (2n = 132) is less than those of other Petromyzontids and Geotriinae but more than that of Mordaciidae [4, 11, 13, 14]. We failed to observe bi-armed chromosomes, suggesting that the lower chromosome number of *L*. *morii* did not derive from centric fusions. This chromosome number is coincidentally close to that predicted for the last common vertebrate ancestor (2n = 136) [14, 31], which is worth further exploration for possible connections. More importantly, based on sequence analysis of several nuclear and mitochondrial DNA fragments, we show that *L*. *morii* appears to have very low heterozygosity (~0.14%), much lower than *P*. *marinus*. This low heterozygosity may indicate a small effective population size for *L*. *morii*, which probably reflects its relatively limited habitat in fresh water. Taken together, our data provide overall background information for further phylogenetic and population genetic research.

## Materials and Methods

### Animals

Adult lampreys (*L*. *morii*) collected from different locations were obtained from local fishermen, Lei Zhao, of the Yalu River, China (around E 126.96°, N 41.80°). Adult *B*. *belcheri* were obtained from local fishermen, Hui Liu, of Zhanjiang, China (E 110.43°, N 21.18°). Zebrafish specimens were obtained from the Fish market of Guangzhou, China (E 113.24°, N 23.09°). Twenty randomly selected individuals of each species were maintained under standard husbandry conditions and supplied with aeration, food and circulating filtered water at the School of Life Science, Sun Yat-Sen University.

No special permits were required for the fishermen to collect the animals. The animal collection and experiments were approved by the Animal Care and Ethics Committee of Sun Yat-Sen University (Permit Number: 20140573). All animals were anesthetized with tricaine methanesulfonate (MS-222) before euthanasia. Lampreys were sacrificed by decollation to obtain erythrocytes and tissues. Adult *B*. *belcheri* were quick frozen and ground in liquid nitrogen for genome extraction. Zebrafish were euthanized by a high dose of anesthesia and tail cutting to collect erythrocytes.

### Single-cell suspension preparation

Fresh chicken blood was obtained from the Poultry market of Guangzhou (E 113.29°, N 23.24°), and EDTA (to 1 mg/mL) was added for anticoagulation immediately. Erythrocytes were centrifugally collected and washed twice in PBS with 50 mM EDTA. The spermary of the lamprey were cut and filtered to obtain sperm. Other tissues (heart, liver, gills, kidneys and intestine) were digested with trypsin at room temperature for 4 hours and then filtered. A single-cell suspension of each tissue was observed with a microscope and diluted to 10^6^ cells per milliliter.

### Flow cytometry

Single-cell suspensions were centrifuged at 300 × *g* for 5 min and resuspended in 10 mM CMF-EDTA [32]. These cell suspensions were adjusted to 75% ethanol and fixed for 30 min on ice. The fixed cells were washed twice in PBS with 0.1% BSA at room temperature and pelleted. Then, the cell pellet was resuspended in propidium iodide staining solution and incubated on ice for 30 min. Fluorescence due to PI staining was measured in a FACSCalibur flow cytometer (Becton Dickinson). Five tissues from each of three adult *L*. *morii* were measured. Erythrocytes from both chicken and zebrafish were used as the internal reference standard.

### Karyotype analysis

Adult *L*. *morii* were injected with 1 mL colchicine solution (0.26%) and maintained in fresh water for 15 hours to arrest cells at metaphase. Then, the individuals were dissected to obtain gills and intestine. The tissues were cut into small pieces and immersed in hypotonic solution (0.46% KCl), which was removed 50 min later. Fresh Carnoy's fluid was added twice, each for 30 min, to fix the cells. To dissociate cells from the epidermis of tissues, 60% acetic acid was added for 5 min. Then, the cell suspensions were dropped onto glass slides, Giemsa-stained after drying, and observed under a high magnification oil microscope. Three randomly chosen adult individuals were tested.

### AFLP protocol

The design and screening principle of the primers and adapters is from Vos et al. [26]. We followed their AFLP protocol with modifications of some parameters to make it suitable for our practical situation. After preliminary experiments, 7 primer combinations were selected to analyze the AFLPs in five randomly chosen adult individuals.

### Amplification of nuclear and mitochondrial genomic fragments

To sample the genetic diversity at the nucleotide level, we cloned one intron from each of five housekeeping genes (ACTL6B, GAPDH, HSP, PGK1 and RPL32). These five genes are conserved between *B*. *belcheri* and lamprey. We also designed a pair of primers to clone the noncoding mitochondrial sequence located between the ND6 and CYTB genes from *L*. *morii*. The primers used to amplify each fragment are listed in Table 4. The amplified fragments were cloned into the pGEM-T vector (Promega) and were sequenced to analyze the differences between individuals. Three randomly chosen adult individuals were used in each test.

**Table 4:** Primers used to amplify genomic and mitochondrial fragments.

Species	Gene	Forward primer (5′ to 3′)	Reverse Primer (5′ to 3′)
*L*. *morii*	*ACTL6B*	CGGAGCGACGCACACTACGG	GGCGATCATGTAGGGAGGGA
	*GAPDH*	TGCGCCAATGTTCGTGATG	CCCTCCACGATGCCGAAGT
	*HSP*	TCGCCCAGTTGATGTCCCTC	CATTTTGCTGGGGTCTGTCA
	*PGK1*	GGGAGACATCTACGTGAACG	AACGGTCTCTCGGGCTTCTC
	*RPL32*	GTCCCCTCGTGAAGCCCAAG	GCCTCACGCGGTTGTCAATG
	NC^a^	ACCAAGCCTAAAGCAGAAAA	TTAGCAGGAGAAGGAAGATC
*B*. *belcheri*	*ACTL6B*	CCCCGCCGGTCTGGAAAAAG	CGCCTGGAAATCTCGCAACA
	*GAPDH*	ACCCATGAAGTCCGAGGA	TGGTGAAGGCTGCATCTG
	*HSP*	AGCCCATCTGGACCCGTAAC	GATCCCCAGACCCAGCTTGA
	*PGK1*	TGGCCTGTTGGGATTCTGCA	CTTCCGGGCATCTCTGACCA
	*RPL32*	GAAATTCATCCGCCATCAGA	TTTTTGCTTCTTGCCCACTG

^a^ noncoding region of lamprey mitochondria genome.
